# In Vitro and In Vivo Relevant Antineoplastic Activity of Platinum(II) Complexes toward Triple-Negative MDA-MB-231 Breast Cancer Cell Line

**DOI:** 10.3390/pharmaceutics14102013

**Published:** 2022-09-22

**Authors:** Leide Laura Figueiredo Maciel, Marina Barreto Silva, Rafaela Oliveira Moreira, Ana Paula Cardoso, Christiane Fernandes, Adolfo Horn, João Carlos de Aquino Almeida, Milton Masahiko Kanashiro

**Affiliations:** 1 Laboratório de Biologia do Reconhecer, Universidade Estadual do Norte Fluminense Darcy Ribeiro, Campos dos Goytacazes 28013-602, RJ, Brazil; 2 Centro Federal de Educação Tecnológica, Nova Friburgo 28635-080, RJ, Brazil; 3 Departamento de Química, Universidade Federal de Santa Catarina, Florianópolis 88040-900, SC, Brazil; 4 Laboratório de Fisiologia e Bioquímica de Microrganismos, Universidade Estadual do Norte Fluminense Darcy Ribeiro, Campos dos Goytacazes 28013-602, RJ, Brazil

**Keywords:** breast cancer cells, platinum(II) complexes, cytotoxicity, apoptosis, antitumor

## Abstract

Two platinum complexes [Pt(HL3)Cl]·H_2_O (**3**) and [Pt(HL4)Cl]·H_2_O (**4**) containing *α-* and *β*-naphthyl groups, respectively, were investigated in more detail in vitro and in vivo for antineoplastic activity. The cytotoxicity activity induced by these platinum(II) compounds against breast cancer (MDA-MB-231 and MCF-7), lung (A549), prostate (PC3), pancreas (BXPC-3), and normal peripheral blood mononuclear (PBMC) cells were evaluated by MTT assay. The cell viability MTT assay showed that complex (**4**) was more cytotoxic to all cancer cell lines tested and less cytotoxic against human PBMC. Therefore, complex (**4**) was selected to further investigate the mechanism of cytotoxic effects involved against MDA-MB-231 cell line (human triple-negative breast cancer). Sub-G1 analysis of the cell cycle showed that this complex induces cell death by apoptosis due to the cell loss of DNA content detected in flow cytometry. The cytotoxic effect induced by complex (**4**) was associated with the capability of the complex to induce mitochondrial membrane depolarization, as well as increase ROS levels and caspase activation, as a result of the activation of both extrinsic and intrinsic apoptosis pathways. Ultrastructural alterations were observed using scanning and transmission electron microscopy (SEM and TEM), such as membrane blebbing, filopodia reduction, empty mitochondrial matrix, and DNA fragmentation. Furthermore, complex (**4**) was tested in an MDA-MB-231 tumor nodule xenograft murine model and demonstrated a remarkable reduction in tumor size in BALB/c *nude* mice, when compared to the control animals.

## 1. Introduction

Breast cancer is the most prevalent type of cancer, with approximately 2.3 million new cases estimated in 2020, and the second leading cause of cancer death in women worldwide [1,2]. It is well known that triple-negative breast cancer, with the absence of estrogen receptor (ER), progesterone receptor (PR), and human epidermal growth factor receptor-type 2 (HER2), is a highly aggressive mammary tumor subtype, responsible for about 12–20% of all breast cancer cases. This type of cancer is associated with younger age of onset, greater metastatic potential, higher incidence of relapse, and lower overall survival rates [3]. Treatment of breast cancer often consists of a combination of therapies such as surgical removal, radiation therapy, and medication (hormonal therapy, chemotherapy, and/or targeted biological therapy) [4].

Development of antitumor metal-based drugs was started with the discovery of cisplatin (CDDP) in 1978, making it one of the most clinically successful antineoplastic drugs used to treat different types of cancers, including testicular, ovarian, melanoma, lymphoma, head and neck, bladder, lung, and several others [5,6]. Later, other platinum-based drugs such as carboplatin and oxaliplatin were produced and approved by the FDA for worldwide clinical practice, and a number of them are in clinical trials studies or in the approval process by the regulatory federal agency as chemotherapeutic drugs [5,7,8]. Although CDDP has been largely used to treat several types of cancers, it has also been reported to cause serious side-effects such as nephrotoxicity, ototoxicity, neurotoxicity and tumor drug resistance, which limit its clinical use [5,6].

Mechanistically, these drugs exert activity via multiple mechanisms, but their most acceptable mechanism involves the generation of DNA lesions with purine bases. The interaction of CDDP with DNA causes distortion of the helical structure, arrest of DNA replication, and blocking of the production of mRNA and proteins, followed by activation of several transduction pathways which finally lead to cell death [5,9]. The dark side of this process is the development of cancer cell resistance to the platinum(II) drugs, because the DNA is easily repaired by the cell DNA repair mechanism [10]. One strategy to avoid this undesirable side-effect is to exploit new complexes with different mechanism of action. Many investigations involving inorganic compounds have focused on the development of new metallodrugs in addition to platinum, such as copper, gallium, ruthenium, cobalt, palladium, and gold complexes, with a broader spectrum of antitumor activity, reduced side-effects, and improved pharmacokinetic properties [9,11,12]. Nonetheless, the search for new Pt-based drugs is an ever-present issue because of the potential of this class of compounds as powerful antineoplastic agents including some of them in preclinical trials [13,14,15,16]. In this context, detailed studies about progress in the research of platinum-based anticancer drugs describing other approaches including nonclassical structures, ligand modifications, incorporation of biomolecules derived assemblies, conjugation with other clinically approved drugs, and targeting drug delivery systems have been reported in numerous works [5,8,16,17,18]. Köberle and Schoch reported promising in vitro and in vivo activity of a variety of new platinum analogs with a different mode of action compared to CDDP tested in cisplatin-unresponsive cancers, such as metastatic colorectal cancer and other solid tumors [19].

**Scheme 1:**
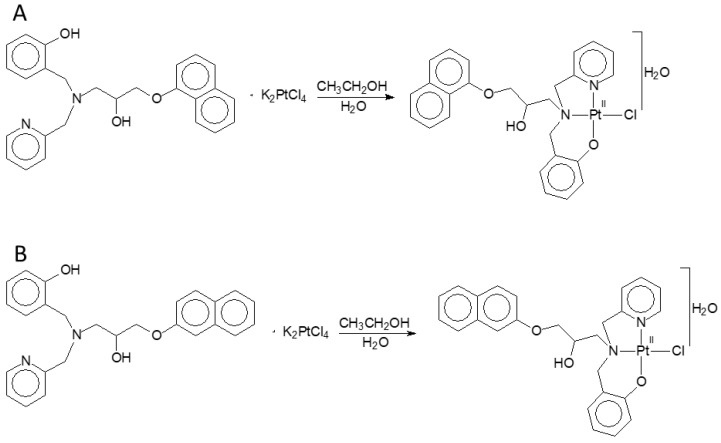
Synthesis of Pt(II)complexes: (**A**) complex (**3**) and (**B**) complex (**4**)**,** based on reference [24].

Our group has been evaluating the cytotoxic activities of a series of coordination compounds containing different metal ions both in vitro and in vivo [20,21,22,23,24]. We previously showed the synthesis, characterization, and cytotoxic activity of four complexes containing platinum(II) as a metallic coordinating center (Scheme 1), where treatment against U937 cell line with complex (**3**) [Pt(HL3)Cl]·H_2_O could induce the activation of both intrinsic and extrinsic apoptotic cascades, as well as perturbations in cell cycle and dysfunction of mitochondrial membrane potential [24]. Aiming to advance this field, we decided to investigate the antineoplastic activity of the Pt(II) complexes (**3**) and (**4**), since the higher activities exhibited by these complexes seem to be related to the presence of the α- and β-naphthyl groups in the ligand structure [24]. Therefore, in these studies, we selected a panel of five cancer cells (MDA-MB-231, MCF7, A549, PC-3, and BXPC-3) and normal cells (PBMC); then, after identifying the most cytotoxic complex, we analyzed its effects on the underlying mechanisms of cell death. Complex (**4**) [Pt(HL4)Cl]·H_2_O, where H_2_L4 = 1-[2-hydroxybenzyl(2-pyridylmethyl)amino]-3-(2-naphthyloxy)-2-propanol), revealed the lowest IC_50_, exhibiting high cytotoxic activity against metastatic MDA-MB-231 cells driving cell death by apoptosis. Additionally, we also evaluated the antitumor efficacy of complex (**4**) on BALB/c *nude* mice in vivo.

## 2. Materials and Methods

### 2.1. Compounds

The platinum(II) complexes were synthesized and characterized as previously reported, and their structures are presented in Scheme 1 [24]. For in vitro testing, stock solutions (20 mM) of the platinum(II) complexes and of the ligands were prepared in dimethyl sulfoxide (DMSO). The structure of the compounds was preserved in DMSO solution (see Supplementary Materials). Cisplatin (Sigma-Aldrich, St. Louis, MO, USA) (5 mM) was diluted in sterile ultrapure water. All solutions were stored at −20 °C. To reach the desired concentrations, the stock solutions were diluted in cell culture medium DMEM-F12 (Gibco, BRL).

### 2.2. Cell Lines

Breast neoplastic (MDA-MB-231 and MCF-7), lung (A549), prostate (PC3), and pancreas (BXPC-3) cell lines were obtained from the Cell Bank of Rio de Janeiro (Federal University of Rio de Janeiro, Rio de Janeiro, Brazil). Normal peripheral blood mononuclear cells (PBMCs) were isolated from the venous blood of healthy volunteers and used as normal cell control. Cancer cell lines were cultured routinely in DMEM-F12 (Dulbecco’s modified Eagle’s medium) and PBMCs were cultured in RPMI 1640 medium (Roswell Park Memorium Institute medium) (Gibco, Carlsbad, CA, USA). Both culture media were supplemented with 10% fetal bovine serum and gentamicin (Gibco, BRL) (20 mg/mL). Cell cultures were maintained at 37 °C in a humidified atmosphere containing 5% CO_2_ (Forma, Thermo Scientific, Inc., Model 3159S/N 33808-427; Waltham, MA, USA). When the cells reached 70–80% confluence, they were detached using 0.25% trypsin and 0.2% EDTA solution, and the culture media were changed. Cell number was established by hemocytometry count, and dead cells were excluded by trypan blue dying [25].

### 2.3. Cell Viability Assay

The cytotoxic effects of the platinum(II) complexes, the ligands, and the metallic salt (K_2_[PtCl_4_]) on cell viability were evaluated using the colorimetric 3-(4,5-dimethyl-2-thiazolyl)-2,5-diphenyl-2H-tetrazolium bromide (MTT) assay, as described by Mosmann [26,27]. CDDP was used as a positive control. A cell concentration of 2 × 10^5^ cells/mL was plated 100/well and treated with different concentrations of the platinum(II) complexes and cisplatin (3.1, 6.2, 12.5, 25, 50, and 100 μM) in DMEM-F12 supplemented culture medium, followed by incubation for 48 h at 37 °C. After treatment, 20 μL of MTT stock solution (5 mg/mL) was added to each well, and the cells were incubated for 4 h at 37 °C. Formazan crystals, produced by viable cells, were dissolved in 100 μL of isopropanol/HCl solution, and their absorbance was determined by a spectrophotometer microplate reader (Epoch™, BioTax instruments, Inc.) at a wavelength of 570 nm. To verify the interference of ROS generation on cell viability, MDA-MB-231 cells were pretreated with the antioxidant N-acetyl-L-cysteine (NAC; 5 mM) (Sigma-Aldrich) 2 h before the addition of complex (**4**). IC_50_ values (the concentration of the compound that causes 50% of cell proliferation inhibition) were obtained from dose–response curves using GraphPad Prism Software version 5.01. The experiments were performed in triplicate.

### 2.4. Apoptosis Evaluation by Cell Cycle Analysis

To evaluate the apoptosis in sub-G1 populations by cell cycle analysis, the MDA-MB-231 cells were seeded at 5 × 10^5^ cells/mL in 12-well plates and treated with 2 × IC_50_ (16 μM) of complex (**4**) and CDDP (126 μM) and incubated for 24 h at 37 °C. After the incubation period, the cells were harvested, centrifuged at 700× *g* for 5 min, and washed twice with cold phosphate-buffered saline (PBS). The cells were then fixed in 70% ethanol for 30 min at 4 °C. The pellets were washed twice with PBS and finally resuspended in 500 µL of PI staining solution (50 µg/mL PI, 1 mg/mL RNAse and 0.2% Triton X-100) for 30 min at room temperature (RT) in the dark. The cell cycle analysis to measure the cell DNA was performed using a flow cytometry (FACS Calibur ™, BD Sciences, Franklin Lakes, NJ, USA), and the apoptotic cell with low DNA content was quantified in the sub-G1 peak of the histograms by recording 10,000 events for each sample. Data were analyzed by WinMDI version 2.9 (The Scripps Institute, San Diego, CA, USA) software.

### 2.5. Determination of Mitochondrial Membrane Potential

The mitochondrial membrane depolarization (∆Ψm) was evaluated using JC-1 staining solution, a cationic dye that accumulates in polarized mitochondria. MDA-MB-231 cells were seeded at 5 × 10^5^ cells/mL in 12-well plates, treated with 2 × IC_50_ of complex (**4**) and CDDP, and incubated for 24 h at 37 °C. Following treatment, the cells were harvested, centrifuged at 700× *g* for 5 min, washed twice in PBS, then stained with JC-1 (25 μg/mL), and incubated at 37 °C for 15 min. Cell pellets were resuspended in fresh PBS, and fluorescence of the mitochondria transmembrane potential was immediately quantified in the FL-1 channel by flow cytometry (FACS Calibur ™, BD Sciences) employing WinMDI version 2.9 software. Data acquisition was determined by recording 10,000 events for each sample.

### 2.6. Measurement of Reactive Oxygen Species (ROS)

To evaluate the involvement of the oxidative stress in the mechanism of cell death induced by complex (**4**) the fluorescent probe 2′,7′-dichlorofluorescein diacetate (H_2_DCFDA) (Sigma-Aldrich) was used to measure ROS production in the cells. MDA-MB-231 cells were seeded at 5 × 10^5^ cells/mL, treated with 2 × IC_50_ of complex (**4**) or CDDP, and incubated for 24 h at 37 °C. In some experiments, cell pretreatment with 5 mM N-acetyl-L-cysteine (NAC), a ROS inhibitor, for 2 h before the addition of complex (**4**) was performed. The cells were then harvested and centrifuged at 700× *g* for 5 min, and the pellets were stained with H_2_DCFDA (2.5 μM/mL) for 30 min at 37 °C. Immediately after, ROS production was measured by flow cytometry (FACS Calibur ™, BD Sciences) employing WinMDI version 2.9 software. Data acquisition was determined by recording 10,000 events for each sample. The cells incubated with H_2_DCFDA probe were also analyzed under an optical microscope (Axiovison A2, Zeiss, Jena, Germany) equipped with a set of fluorescent filters (excitation wavelength between 450–490 nm and 500 nm emission). The experiments were performed in triplicate.

### 2.7. Caspases Activity Assay

The activities of caspases 3, 6, 8, and 9 were determined using the substrates Ac-DEVD-pNA, VEID-pNA, IETD-pNA, and LEHD-pNA (Sigma-Aldrich), specific for the respective caspases, according to the manufacturer’s instructions. Briefly, MDA-MB-231 cells were seeded at 3–5 × 10^6^ cells/mL in 96-well plates, treated with 2 × IC_50_ of complex (**4**) or with CDDP, and incubated for 12 and 24 h at 37 °C. Next, the cells were harvested and washed twice with PBS, and the pellets were resuspended in 100 μL of lysis buffer (1% Triton X-100, 0.32 M sucrose, 5 mM EDTA, 10 mM Tris-HCl (pH 8.0), 2 mM DTT, 1 mM PMSF, 1 g/mL aprotinin, and 1 mg/mL leupeptin) and left on ice for 10 min. The sample was submitted to strong agitation to disrupt cellular membranes and centrifuged at 15,000 rpm in a microcentrifuge for 10 min at 4 °C and then the supernatants were collected in new tubes. A BCA protein assay kit was used to determine protein concentration, and 50 μL aliquots of the supernatant were placed in a 96-well plate containing reaction buffer (Invitrogen™). A volume of 5 μL of each substrate was added, and the plate was incubated for 1 h at 37 °C. Optical densities were measured in a spectrophotometer (EPOCH™, BioTek^®^ Instruments, Inc., Winooski, VT, USA) at a wavelength of 405 nm. The absorbance of caspase activation of treated cells was compared with an uninduced control sample.

### 2.8. Morphological Assessment by Hoechst 33342/MitoTracker Double Staining

Morphological changes were evaluated using Hoechst 33,342 (Thermo Scientific, Waltham, MA, USA) and MitoTracker fluorescent dyes (Invitrogen, Thermo Scientific, Waltham, MA, USA), a blue-fluorescent stain specific for DNA, which passes through the cell and a red-fluorescent dye that stains mitochondria in live cells, whose accumulation is dependent upon membrane potential, respectively. MDA-MB-231 cells were seeded at 5 × 10^5^ cells/mL in 12-well plates, treated with 2 × IC_50_ of complex (**4**) or with CDDP, and incubated for 12 h at 37 °C. Subsequently, the cells were harvested and centrifuged at 700× *g* for 5 min, and the pellets were resuspended with the Hoechst 33342 (1 μM/mL) and MitoTracker (100 nM/mL) fluorescent dyes and incubated for 15 min at 37 °C. Nuclear and mitochondrial morphology of the cells were examined under an optical microscope (Axiovison A2, Zeiss, Jena, Germany) equipped with a set of fluorescent filters, and images were captured at 20× and 40× magnification.

### 2.9. Transmission Electron Microscopy

Ultrastructural analyses were detected using scanning (SEM) and transmission electron microscopy (TEM). MDA-MB-231 cells were seeded at 5 × 10^5^ cells/mL in a 25 cm^2^ culture flask, treated with 2 × IC_50_ of complex (**4**) and CDDP and incubated for 4, 8, and 12 h at 37 °C. After treatment, the cells were harvested, centrifuged at 700× *g* for 5 min, and washed twice with PBS; then, the pellets were fixed in 2.5% glutaraldehyde in 0.1 M sodium cacodylate buffer (pH 7.4) for 2 h at RT. The cells were washed three times with PBS and post-fixed for 20 min in a 1:1 solution of osmium tetroxide (1%) and potassium ferricyanide (0.8%). The samples were then prepared for SEM and TEM following routine procedures, as reported previously [22].

### 2.10. Determination of Acute Toxicity of Complex (**4**)

BALB/c *nude* mice, 6–8 weeks old, bred in the facilities of the UENF central vivarium were housed in ventilated cages at controlled temperature and humidity in a 12 h light/dark cycle, with free access to feed and water. Studies were approved by the Animal Ethics Use Committee of the Universidade Estadual do Norte Fluminense (Campos dos Goytacazes, Rio de Janeiro, Brazil) under protocol number 349. Groups of four female BALB/c *nude* mice between 20 g and 25 g was used to determine the acute toxicity of complex (**4**) dissolved in ultrapure water. Concentrations of 1, 10, and 100 mg/kg of the complex (**4**) were injected into the peritoneal cavity using insulin syringes with a hypodermic needle (0.45 mm × 13 mm, Nipro Medical Corporation do Brasil, Sorocaba, Brasil). The control group received only ultrapure water. The animals were observed closely for at least 4 h post injection and at 24 h intervals for 15 days. After this period, the median lethal dose (LD_50_), a dose required to kill half the inoculated animals, was established. Survival curves were constructed using the Kaplan–Meier method.

### 2.11. Analysis of Antitumor Efficacy In Vivo

Groups of female BALB/c *nude* mice (six mice per group) were subcutaneously inoculated with 3 × 10^6^ MDA-MB-231 cells in a volume of 50 μL of PBS in the back of the animals. This experiment was performed according to methodology described by Maciel et al. [22]. Briefly, BALB/c *nude* mice bearing tumor nodules (±3 mm) were divided into three groups of six animals. Complex (**4**) 9.2 mg/mL was diluted in PBS containing 20% DMSO, and 100 μL was i.p. injected for animals weighing 20 g. The volume was adjusted for animals with different weights, ranging between 20 and 25 g. For CDDP, 0.66 mg/mL was prepared in PBS, and i.p. injection was performed the same way as for complex (**4**). The treated group received 46 mg/kg of complex (**4**), while the CDDP control group received 3.3 mg/kg, with both concentrations corresponding to 50% of the LD_50_. Only vehicle solution was injected into the negative control group of mice. Four doses of the treatment were administered to the animals in 1 week intervals. Measurements of the tumor size was performed every 3 days during the experiment period using a Mitutoyo caliper.

### 2.12. Statistical Analysis

Results were expressed as the mean ± standard deviation (mean ± SD) for in vitro and in vivo studies. For statistical analysis, the ANOVA test was used, followed by the Bonferroni post-test. All data were performed using GraphPad Prism, version 5, for Windows. The survival curve and tumor volume were estimated using the Kaplan–Meier method. Values of *p* < 0.05 were considered statistically significant (* *p* < 0.05; ** *p* < 0.01; *** *p* < 0.001).

## 3. Results

### 3.1. Assessment of Cell Viability by MTT Assay

The in vitro cytotoxicity of platinum(II) complexes (**3**) and (**4**), ligand H_2_L4, and the reference metallodrug cisplatin were evaluated against a panel of five human cancer cell lines (MDA-MB-231, MCF-7, A549, PC3, and BXPC-3) using the colorimetric MTT assay. In general, complexes (**3**) and (**4**) were effective in reducing the proliferation of all the tested cell lines. As shown in Table 1, complex (**4**) showed a more pronounced cytotoxic effect on breast cancer MDA-MB-231 cell line, with the IC_50_ value eightfold lower than cisplatin (IC_50_ 8.1 ± 1.1 µM vs. 63.1 ± 1.2 µM). In contrast, the free ligand H_2_L4 treatments at the highest concentrations of 100 μM did not affect the viability of any of the cell lines probed. To verify the selectivity of complex (**4**), we also tested the compound against normal peripheral blood mononuclear cells (PBMC). Interestingly, complex (**4**) presented IC_50_ values of 42.2 μM, being comparable to that determined for CDDP 44 ± 2 μM (Table 1). The selectivity index (SI) is defined as the IC_50_ ratio of cytotoxicity of normal cells to cancer cells, being selective when the ratio is greater than two [28]. Thus, complex (**4**) also showed the best selectivity index toward the MDA-MB-231 cell line (SI = 5.2), which is at least seven times higher than cisplatin (SI = 0.7), suggesting that complex (**4**) has a selective cytotoxic effect for neoplastic cells, as shown in Table 2. Those data analyses indicate that the complex (**4**) and the breast cancer MDA-MB-231 cell line justify further studies to unravel the mechanism of cell death induced by the compound in vitro and in the in vivo systems.

**Table 1:** Cell cytotoxicity induced by the platinum complexes determined by MTT assay.

IC_50_ (μM)						
Compounds	MDA-MB-231	MCF-7	A549	PC-3	BXPC-3	PBMC ^a^
Complex (**3**)	14.4 ± 1.1	20.5 ± 1.1	35.3 ± 1	23 ± 1.1	16.3 ± 1.1	22.0 ± 1.0
Complex (**4**)	8.0 ± 1.1	16.2 ± 1.2	18 ± 1.1	14.4 ± 1.2	13.7 ± 1.1	42.0 ± 1.0
(H_2_L4)	>100	>100	>100	>100	>100	>100
CDDP	63 ± 1.2	20.7 ± 1.1	70 ± 1.2	45.6 ± 1.0	12.7 ± 1.2	44.0 ± 2.0

Data are expressed as the mean ± SD of three independent experiments, each in triplicate. ^a^ IC_50_ values reported by [24].

**Table 2:** Selectivity index of complex(II) and cisplatin for cancer cells. SI = IC_50_ for the noncancer line (PBMC)/IC_50_ for the cancer cell line.

Selectivity Index (SI ^b^)					
Compounds	MDA-MB-2311	MCF-7	A549	PC-3	BXPC-3
Complex (**3**)	1.5	1.1	0.6	0.95	1.3
Complex (**4**)	5.2	2.6	2.3	3	3.1
CDDP	0.7	2.1	0.6	1	3.5

^b^ An SI value >2 indicates high selectivity [29].

### 3.2. Complex (4) Induces Apoptosis in Human Breast Cancer Cells

**Figure 1:**
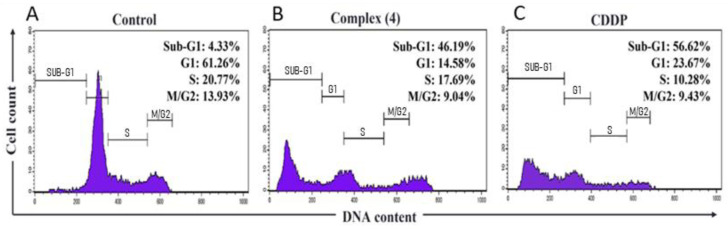
Cell cycle analysis by flow cytometry of MDA-MB-231 cells after 24 h of incubation and stained with propidium iodide (PI). Histograms representative of cell cycle progression show the percentage distribution of cells in different cell cycle phases. Cells with hypodiploid DNA content were measured by the sub-G1 fraction. (**A**) Control cells. (**B**) Cells treated with 2 × IC_50_ of complex (**4**). (**C**) Cells treated with 2 × IC_50_ of CDDP. Each experiment per sample was determined by recording 10,000 events. Data are representative of three independent experiments.

We evaluated the induction of apoptosis after the treatment of MDA-MB-231 cells with complex (**4**) and CDDP by measuring the cell DNA content in the sub-G1 region in the cell cycle analysis using a flow cytometer. Cells were stained with propidium iodide (PI), and the cell cycle progression was analyzed. As shown in Figure 1, in the untreated control group, most of the cells were in the G0/G1 and S phases, and the cell population in the sub-G1 region was only 4.3%. When the breast cancer cells were treated with 2 × IC_50_ of complex (**4**) for 24 h, a marked accumulation of cells in the sub-G1 region (46.2%) and a reduction in the number of cells in the G0/G1 phase was observed. After treatment with cisplatin, 57% of the MDA-MB-231 cells were in the sub-G1 region. The loss of the DNA content by the cells in the sub-G1 regions indicates cell death by apoptosis.

### 3.3. Complex (**4**) Decreases the Mitochondrial Transmembrane Potential

**Figure 2:**
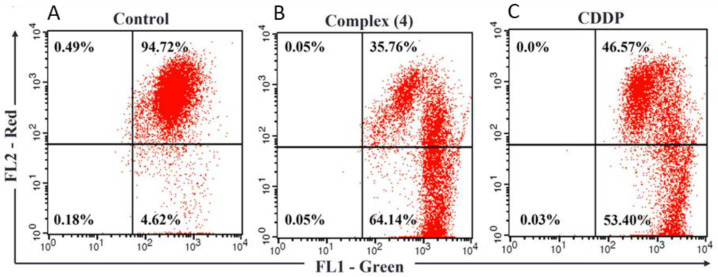
Mitochondrial transmembrane potential (∆Ψm) assay of MDA-MB-231 cells after 24 h of incubation. Cells were stained with JC-1 and analyzed using flow cytometry. Each dotplot shows the percentage of cells with ∆Ψm loss, as indicated by the cell change from red fluorescence to green fluorescence. (**A**) Control cells. (**B**) Cells treated with 2 × IC_50_ of complex (**4**). (**C**) Cells treated with 2 × IC_50_ of CDDP. Data are representative of three independent experiments.

Mitochondria are among the most important organelles that can regulate cell death, as mitochondrial membrane permeabilization generally precedes the activation of the intrinsic apoptosis pathway [30]. Mitochondrial transmembrane potential (∆Ψm) loss is a hallmark of mitochondrial dysfunction [31]. To elucidate if the cytotoxic effects of complex (**4**) are associated with mitochondrial dysfunction, we evaluated the ∆Ψm in MDA-MB-321 breast cancer cells via flow cytometry using the lipophilic JC1 probe. As presented in Figure 2, MDA-MB-231 cells emitting green fluorescence were observed in 64.1% of cells treated with 2 × IC_50_ of complex (**4**) for 24 h, indicating the depolarization of the mitochondrial membrane. In the 2 × IC_50_ of the CDDP treatment group, 53.4% of MDA-MB-231 cells emitting green fluorescence were observed (Figure 2). Comparing both compounds, complex (**4**) showed greater effectiveness than CDDP to induce the decrease in ∆Ψm. Untreated control cells showed 94.7% of red fluorescence, resulting from the aggregated form of JC-1 stain, with only 4.6% emitting green fluorescence.

### 3.4. Alteration in Nuclear and Mitochondrial Morphology

**Figure 3:**
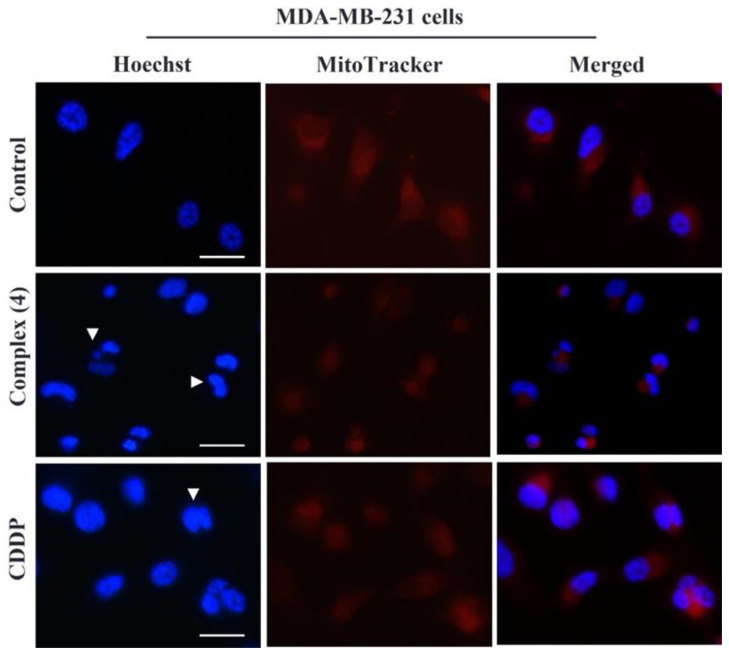
Morphological changes of MDA-MB-231 cells double-stained with Hoechst/MitoTracker examined under a fluorescence microscope after 12 h of incubation. Control cells. Complex (**4**)—cells treated with 2 × IC_50_ of complex (**4**). CDDP—cells treated with 2 × IC_50_ of cisplatin. Chromatin condensation and the pyknotic/fragmented nucleus of apoptotic cells are indicated by arrowheads. Data are representative of two independent experiments. Bars = 20 μm.

We carried out nuclear and mitochondrial integrity evaluation by fluorescence microscopy using Hoechst/MitoTracker double staining. In the untreated control cells, we observed an organized cellular structure where the nuclei were homogeneous and blue-stained; in contrast, the mitochondria were stained in red, showing a thin layer on the periphery of the cells (Figure 3). For MDA-MB-231 breast cancer cells, after 12 h of incubation with 2 × IC_50_ of complex (**4**) and CDDP, several morphological changes were observed. Cellular fluorescence patterns revealed typical morphology of apoptotic cells after treatment with complex (**4**), such as nuclear shrinkage, lunate morphology of chromatin, or bright-blue fragments of DNA. Furthermore, the decline in red fluorescent intensity dispersed in the cytoplasm indicated that ∆Ψm was reduced in the treated cells, as compared with a balanced mitochondrial network in untreated cells, evidencing that the complex caused abnormality of cell morphology. DNA fragmentation and binucleation were also detected in MDA-MB-231 cells when treated with CDDP, similar to the complex (**4**)-treated group (Figure 3).

### 3.5. Complex (**4**) Induces Cellular Cytotoxicity by ROS Production

**Figure 4:**
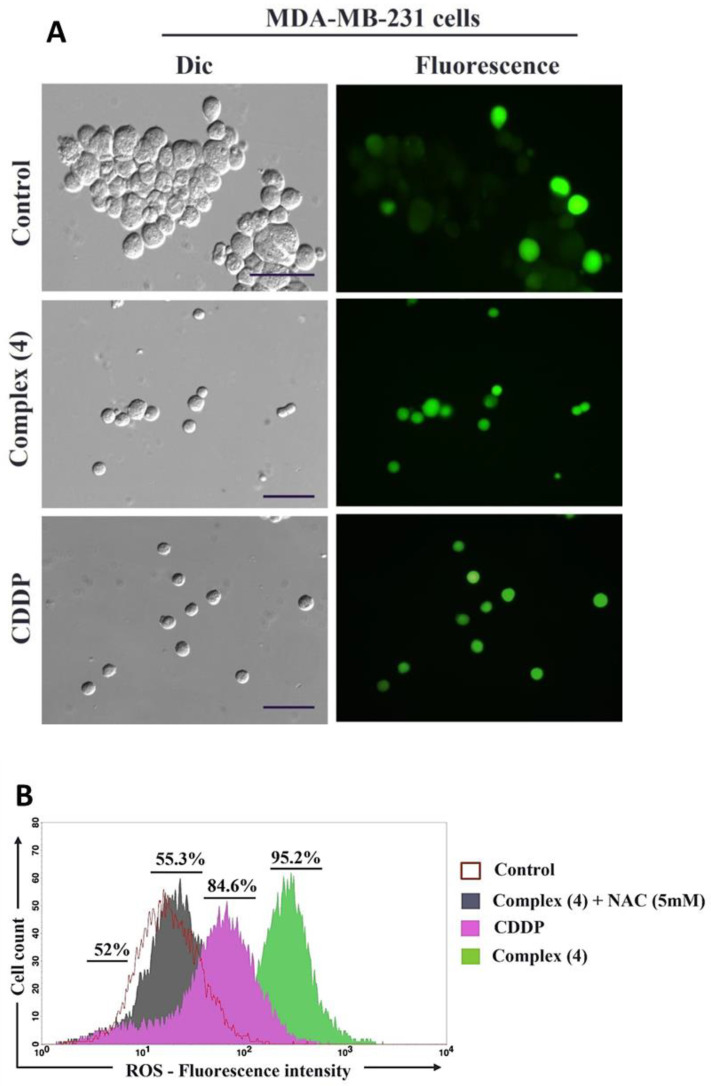

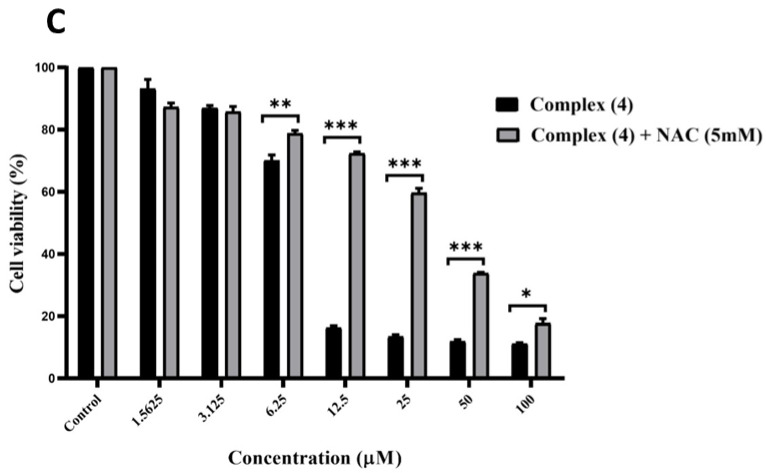
The levels of ROS detected in MDA-MB-231 cells after treatment with 2 × IC_50_ of the compounds for 24 h using the H_2_DCFDA fluorescent probe and analyzed by light microscopy and flow cytometry. (**A**) Representative images show increased ROS generation in MDA-MB-231 cells after treatment with compounds. Control cells were treated only with H_2_DCFDA probe. Bars = 20 μm. (**B**) Quantitative analysis of ROS production by flow cytometry and ROS inhibition of ROS production with NAC 5mM. (**C**) Cellular cytotoxicity evaluation by MTT assay after 48 h of treatment with complex (**4**) in the presence or absence of NAC. Data represent the mean ± SD (*n* = 3). * Indicates statistical difference (* *p* < 0.05, ** *p* < 0.01, and *** *p* < 0.001).

Oxidative stress has been reported as one of the main factors that trigger the deleterious actions of metal-based drugs [32]. High productions of ROS are typically linked to potential mitochondrial membrane damage followed by cell death. To verify whether complex (**4**) triggers ROS generation, breast cancer cells were incubated with 2 × IC_50_ of the compounds for 24 h. Cellular ROS production was then measured using an H_2_DCFDA probe by flow cytometry and fluorescence microscopy. As shown in Figure 4A, fluorescence microscopy analysis clearly showed many more positive cells with stronger fluorescent intensity in MDA-MB-231 cells treated with complex (**4**) and CDDP, suggesting a high capability to induce ROS generation by both compounds. Quantitative analysis by flow cytometer showed that complex (**4**) and CDDP induced 95.2% and 84.6% cell ROS production, respectively, while 52% of control untreated MDA-MB-231 cells produced constitutive ROS (Figure 4B). When the MDA-MB-231 cells were treated with complex (**4**) and the antioxidant (NAC; 5 mM), a marked reduction in ROS production was observed (55.3%), with values similar to the control cells (Figure 4B). We also evaluated the involvement of ROS in the cellular cytotoxicity induced by complex (**4**). Figure 4C shows that the inhibition of ROS by the use of NAC resulted in a strong increment in cell viability, with an IC_50_ increase from 8 ± 1.1 μM to 30.5 ± 1.1 μM.

### 3.6. Effect of Complex (**4**) Treatment on Caspases Activation

**Figure 5:**
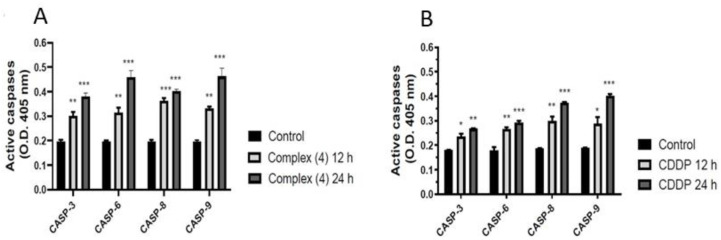
Determination of active caspases in MDA-MB-231 cells. Activation of caspases 3, 6, 8, and 9 was determined after 12 and 24 h of treatment with 2 × IC_50_ of complex (**4**) or with cisplatin. (**A**) MDA-MB-231 cells treated with complex (**4**); (**B**) MDA-MB-231 cells treated with CDDP. Values are means ± SD from two independent experiments performed in triplicate. * *p* < 0.05, ** *p* < 0.01, and *** *p* < 0.001 versus untreated control.

To investigate the apoptosis pathway activated by complex (**4**), we evaluated the caspase activities in MDA-MB-231 after treatment with the compound and CDDP. To this end, we evaluated the activation of caspase-8 and caspase-9, indicative of extrinsic (via death receptor) and intrinsic apoptosis (via mitochondrial), respectively, and activation of effector caspases (3 and 6), proteins closely associated with the execution of apoptosis. Our results show that treatment of MDA-MB-231 cells with 2 × IC_50_ of complex (**4**) after 12 and 24 h resulted in a significant increase in active caspase levels compared to untreated cells (Figure 5A). CDDP used as a positive control, was also able to activate all tested caspases (Figure 5B), thus leading to the activation of downstream components of both intrinsic and extrinsic apoptotic cascades.

### 3.7. Complex (**4**) Affects the Ultrastructure of Breast Cancer Cells

**Figure 6:**
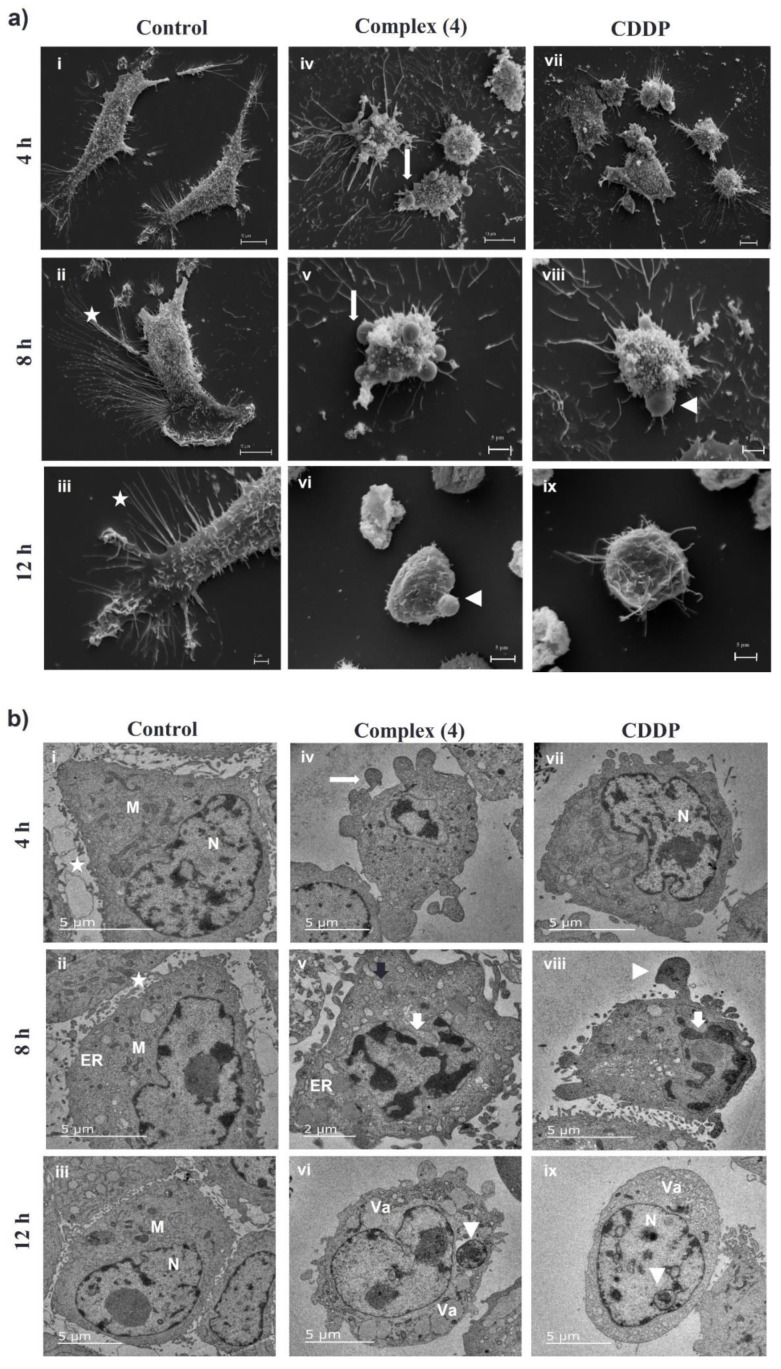
Ultrastructural analysis of MDA-MB-231 cells treated with 2 × IC_50_ of complex (**4**) and cisplatin after 4, 8, and 12 h of incubation in comparison to the controlled group. (**a**) The top panel shows representative SEM micrographs of the compounds’ effect on the surface of the MDA-MB-231 cell line. (**b**) The bottom panel shows representative TEM micrographs of the compounds’ effect on the intracellular organelles of MDA-MB-231 cell line. Numerous villi are shown (star) on the cell surface. Cells with intense membrane blebbing (thin white arrow). The release of apoptotic bodies can be seen (arrowhead). Empty mitochondrial matrix (black arrow). Cells have a cytoplasm diffuse vacuolization and contain a reduced number of organelles (Va). (M) Mitochondria; (ER) endoplasmic reticulum; (N) nucleus. Chromatin condensation is indicated by the white arrow.

MDA-MB-231 cell line treatment with 2 × IC_50_ of complex (**4**) and with cisplatin for 4, 8, and 12 h of incubation was investigated at an ultrastructural level by SEM (Figure 6a) and TEM (Figure 6b). Untreated control group cell micrographs revealed a surface structure containing microvilli with numerous membrane-bound filopodia, evidencing their viability and potential for proliferation and invasion, and the cell body exhibited an elongated spindle shape, as shown in Figure 6(ai–aiii). Cellular organelles such as mitochondria and endoplasmic reticulum (ER), when observed at the ultrastructure level, appeared in large numbers in the cytoplasm and distributed in a homogeneous form, indicating a good cell viability, as well as nuclear chromatin (Figure 6(bi–biii)). In the first 4 h of treatment with complex (**4**), cell alterations typical of the apoptotic process were detected, such as membrane blebbing, chromatin condensation, and a retraction in the cell surface filopodia, which can lead to loss of adhesion to the matrix (Figure 6(a,biv)). In 8 h of treatment with complex (**4**), drastic changes were observed in the cells such as intense membrane blebbing (Figure 6(av)), as well as compaction of the chromatin in the periphery of the nucleus, fragmentation of DNA, and ER enlargement, as shown in Figure 6(bv). Moreover, in Figure 6(bv), the mitochondrial matrix is rarefied, becoming more electron-lucent in the image by TEM in some organelles, suggesting an empty matrix (indicated by black arrow), and reinforcing that the mitochondria are a target of complex (**4**). In the prolonged treatment after 12 h, cells underwent profound modifications in shape when compared to the control group. The cell features were reduced in size and rounded in shape, with the total absence of filopodia (Figure 6(avi)); cytoplasmic vacuolization was evident (Va), ultimately leading to disintegration of organelles and cell death (Figure 6(bvi)). During the period of 4–12 h of observations, no morphological alterations were observed in the plasma membrane; all these processes were consistent with apoptosis activation and cell stress. On the other hand, CDDPtreated cells showed mild alterations when compared to the MDA-MB-231 cell line incubated with complex (**4**), leading us to believe that complex (**4**) induces cytotoxic activity more rapidly when compared to a standard drug. Changes in nuclear morphology, increased margination, and condensation of the genetic material (white arrow) (Figure 6(aviii)) were the main morphological changes observed in MDA-MB-231 cells treated with CDDP. Additionally, formation of apoptotic bodies (arrowhead) was also observed (Figure 6(a,bviii)). These results are in agreement with alterations shown by the Hoechst/MitoTracker probe analysis.

### 3.8. Complex (**4**) Reduces Tumor Growth in a Murine Model of Breast Cancer

**Figure 7:**
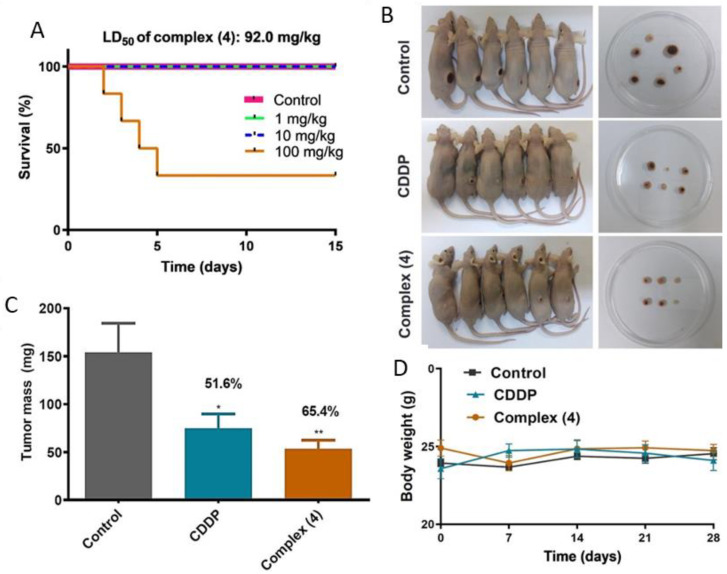
In vivo efficacy of complex (**4**) in the tumor growth inhibition in Balb/c *nude* mice bearing MDA-MB-231 breast tumors (*n* = 6 in each group). Animals were treated i.p. with 50% of the LD_50_ of complex (**4**) (46 mg/kg) or cisplatin (3.3 mg/kg) in four doses at 1 week intervals. (**A**) Determination of acute lethal dose; (**B**) animals and dissected tumor nodules; (**C**) mean total weight of the dissected tumors after the end of treatment; (**D**) body weight loss curves during the treatments. Data were analyzed by ANOVA/Bonferroni and expressed as the mean ± SD; a significant difference was taken as * *p* < 0.05 and ** *p* < 0.01 when comparing the treated and control groups.

We further evaluated the in vivo anticancer efficacy of Pt(II) complex in MDA-MB-231 tumor-bearing mice. To define the initial concentration for mice treatments, we determined the acute lethal dose (LD_50_) of complex (**4**) in BALB/c *nude* mice. Figure 7A shows the LD_50_ at 92.6 mg/kg for BALB/c *nude* mice. Subsequently, mice treatment with 50% of LD_50_ of each compound was performed—46 mg/kg of complex (**4**) and 3.3 mg/kg of cisplatin [33]. Groups of six female BALB/c *nude* mice bearing nodules of breast cancer cells were inoculated via the intraperitoneal route with each of the compounds weekly for up to 4 weeks. Three days after the last dose, the animals were euthanized, and tumors were excised and weighed. Figure 7B shows the in vivo antitumor effects of complex (**4**) on the human breast cancer murine model. Tumor mass (mg) can be observed in Figure 7c, with tumor weights of 153.6 ± 48, 53.2 ± 16, and 74.3 ± 24 mg for control, treatment with complex (**4**), and treatment with CDDP, respectively. In this sense, complex (**4**) significantly inhibited tumor nodules by 65.4% at the end of treatment (** *p* < 0.01). Likewise, tumor weight in the cisplatin-treated group was also decreased by 51.6% when compared with the control group (* *p* < 0.05) (Figure 7C). Although complex (**4**) seemed to be slightly better than CDDP, the difference between the treatments was not statistically significant. In all groups, animals were not affected with serious side-effects, as monitored by body weight (Figure 7D). Our in vivo results agree with the in vitro data showing that complex (**4**) exerted a cytotoxic effect against the MDA-MB-231 breast cancer cell line.

## 4. Discussion

In our previous paper [24], we showed the synthesis, physicochemical characterizations, and general biological activity of complex (**3**) and (**4**); in this work, we first investigated the potential anticancer activity these platinum(II) complexes and compared their in vitro cytotoxicity with CDDP in a panel of five cell lines derived from different human cancers. Similar results were observed for complexes containing Cu(II) and Pt(II), as previously reported by us [22,23,24]. We also showed the striking difference in cytotoxicity of complex (**4**) in contrast to CDDP, the chemotherapeutic reference (IC_50_ 8.1 ± 1.1 µM vs. 63.1 ± 1.2 µM), which was at least eightfold lower for the MDA-MB-231 cancer cell line. Furthermore, we investigated the effect of complex (**4**) on the viability of normal peripheral blood mononuclear cells (IC_50_ = 42.2 ± 1.1 μM), showing that the complex was more active against MDA-MB-231 cells. The selectivity index (SI = 5.2) indicates that complex (**4**) had a selective effect on the MDA-MB-231 cell line. Thus, since complex (**4**) presented a promising antiproliferative effect and selectivity against cancer cells, this motivated us to investigate more deeply the mechanism of action of the compound on MDA-MB-231 cells (human triple-negative breast cancer) through in vitro and in vivo assays.

The evasion of apoptosis is a crucial feature of carcinogenesis and contributes to resistance to antineoplastic drugs. Thus, targeting signaling pathways that activate apoptotic processes is an attractive strategy for cancer therapy [34]. In general, platinum-based compounds are considered as cytotoxic drugs which kill cancer cells by damaging DNA. Several anticancer compounds exert their cytotoxicity by arresting the cell cycle and inducing apoptosis [9,35]. In the current study, we showed the increase in the number of cells in the sub-G1 peak (46.2%) when MDA-MB-231 was incubated with complex (**4**), while, for CDDP, 57% was observed in the same region of the histogram (Figure 1). In cell cycle analysis using flow cytometry, the sub-G1 region of the histogram corresponds to low-DNA-content cells, which is associated with the late stages of apoptosis [36].

The intrinsic (mitochondrial) and extrinsic (death receptor) apoptosis pathways can be stimulated by many different activators [36]. The activation of caspase-8 and caspase-9 is well correlated with triggering the extrinsic (death receptor) and intrinsic (mitochondrial) apoptosis pathways, respectively, and activation of effector caspases 3 and 6 is associated with the execution pathway of apoptosis. These proteases are critical mediators of the programmed cell death mechanism, which, in turn, cleave cellular substrates, leading to morphological and biochemical changes of apoptosis [36]. Our result shows that complex (**4**) and cisplatin were able to activate all tested caspases, including caspases 8 and 9 in the MDA-MB-231 cell line, when treated with the compounds (Figure 5), thus leading to the activation of downstream components of both extrinsic and intrinsic apoptotic cascades, in agreement with our previous work [24]. Mitochondria play a major role in the early phase of the intrinsic apoptosis pathway. This pathway is associated with increased mitochondrial membrane permeability and the release of proapoptotic molecules such as cytochrome-c into the cytoplasm [36]. Several previous studies showed that anticancer platinum-based drugs induce apoptosis as a consequence of mitochondrial collapse [16,37,38]. Our data demonstrate that complex (**4**) can interfere in the ∆Ψm, increasing the proportion of cells with depolarized mitochondria (Figure 2), confirming that the intrinsic pathway of apoptosis was activated with the involvement of mitochondrial dysfunction. These results are in the same line of evidence for copper(II), iron(III), and cobalt(II) compounds previously published by our group [21,23,39]. Additionally, complex (**4**) and CDDP added to the MDA-MB-231 cell culture showed typical morphological changes observed in the apoptosis process such as chromatin condensation, nuclear fragmentation, and evident mitochondrial structure disruption (Figure 3). Furthermore, mitochondrial damage can be associated with the generation of a high level of ROS, and ROS imbalance can cause cellular dysfunction via oxidative stress, thereby driving the cells to apoptosis death [32,40]. Notably, both compounds increased intracellular ROS generation; however, complex (**4**) was able to induce a higher level of ROS in comparison to CDDP (Figure 4b). Our results suggest that ROS play an important role in the cytotoxic effect of complex (**4**) since this effect was significantly blocked when the cells were pretreated with NAC, a specific ROS inhibitor (Figure 4b,c). In agreement with our results, it is known that metal-based drugs, including CDDP, induce DNA damage mediated by free radicals, resulting in cell death by apoptosis [32,41]. Additionally, our results corroborate several studies based on Cu(II), Ru(II), and Au(III) complexes showing that those metal compounds induce cytotoxic activity through the increment in ROS generation as the underlining mechanism to induce cell death by apoptosis in breast adenocarcinoma cells and ovarian carcinoma, breast cancer cells [42], and prostate cancer cells [43], respectively.

Microscopy analysis is an important tool to detect cellular morphological changes including those that occur during apoptosis, such as cell shrinkage, membrane blebbing, chromatin condensation, and formation of apoptotic bodies [40]. To identify the cellular structures and organelles targeted by complex (**4**) treatment, we used ultrastructural analysis by SEM and TEM of MDA-MB-231 cells after exposure to the compound. SEM analysis was used to verify the cellular surface morphological alterations after treatment with complex (**4**) and cisplatin showing significant changes in the cell membrane, such as membrane blebbing suggestive of apoptosis and the appearance of round cells, which is an indication of cell detachment and loss of invasive features such as filopodia (Figure 6(av–aviii)). Supporting the SEM findings, TEM analysis showed strong changes to cell morphology in comparison to the untreated control, such as nuclear collapse, chromatin condensation, formation of numerous vacuoles, and apoptotic body in MDA-MB-231 cells after exposure to the compounds. Altered mitochondria were observed, and some of them showed an empty matrix (Figure 6(bv)), as well as evidence of endoplasmic reticulum dilation, suggesting the possible participation of this organelle in the apoptotic process (Figure 6(biv–bvi). Hence, our data are consistent with other findings in which apoptosis was the major mechanism of cell death that appeared in breast cancer cells following exposure to metal complexes, where ultrastructural and cell membrane changes, such as irregular protuberances and filopodia retraction, were reported to be closely associated with actin cytoskeleton disorder [4,44]. A cytoskeleton enables the cell to ensure its shape, structural integrity of organelles, and cell motility, in addition to promoting cell–cell or cell–matrix adhesion via their interactions with cadherin and integrins, respectively [45]. Taking together our results, we can hypothesize that complex (**4**) could act by inhibiting the expression of adhesion molecules that mediate the binding of MDA-MB-231 to extracellular matrix proteins. However, the exact mechanism of complex (**4**) that affects the MDA-MB-231 cells remains to be elucidated.

Lastly, consistent with our results, we showed that complex (**4**) maintains antitumoral activity in vivo. We showed a substantial decrease in MDA-MB-231 tumor nodule growth in mice. The demonstration of in vivo antineoplastic activity is an essential step for further clinical trial candidates [46]. In our previous report, we showed that the complex [Cu(L1)Cl]Cl·2H_2_O efficiently inhibits the tumor nodule growth in BALB/c *nude* mice bearing NCI-H460 lung cancer xenografts [22]. Here, we showed that complex (**4**) was less toxic to BALB/c *nude* mice than the standard drug CDDP (LD_50_ 92mg/kg vs. 6.6 mg/kg), and it reduced 65.4% of tumor growth without affecting the body weights of animals when compared to the control group (Figure 7). Overall, our results highlighted the interesting role of Pt(II) complex in MDA-MB-231 cells, suggesting their relevant and potent cytotoxic activity toward the breast cancer cell line, which is worthy of further study.

## 5. Conclusions

Among many studies regarding the cytotoxic effects of platinum compounds on anticancer properties, this work showed the cellular responses to treatment of a Pt(II) complex in MDA-MB-231 cell lines. Complex (**4**) presents IC_50_ values lower than CDDP toward cancer cells and comparable values for peripheral blood mononuclear cells (PBMCs). Morphological and ultrastructural evidence points toward their capacity to impair MDA-MB-231 cell survival by interfering with the DNA and mitochondria, along with possible participation of the endoplasmic reticulum. Complex (**4**) exerts its cytotoxicity by inducing a high production of endogenous ROS, thus decreasing mitochondrial function of the cancer cell line. Moreover, complex (**4**) promoted the activation of the initiator caspases 8 and 9 at the same time, suggesting that it activates both intrinsic and extrinsic apoptotic cell death pathways. We also demonstrated that, in mice bearing MDA-MB-231 tumor nodules, complex (**4**) significantly reduced tumor mass at 65.4% when compared to vehicle-treated control mice.

## 6. Patents

Patent pending at INPI (National Institute of Industrial Properties) BR 10 2016 028580 1—Brazil.

## Supplementary Materials

The following supporting information can be downloaded at: https://www.mdpi.com/article/10.3390/pharmaceutics14102013/s1.
