# Supplementary material for: In Vitro and In Vivo Relevant Antineoplastic Activity of Platinum(II) Complexes toward Triple-Negative MDA-MB-231 Breast Cancer Cell Line
Source: Pharmaceutics. 2022 Sep 22;14(10):2013. doi: 10.3390/pharmaceutics14102013 (PMC9609024; doi:10.3390/pharmaceutics14102013)
Supplement: Supplementary file 1 [file pharmaceutics-14-02013-s001.zip › pharmaceutics-1860411-supplementary.pdf]

Supplementary Material

The study of the stability, in DMSO and in biological medium is important to understand the behavior of the complexes in solution. In order to detect any changes after diluting the platinum complexes in DMEM (biological medium), electronic spectra of both complexes were also obtained in DMSO and in DMEM medium, for comparison. As the compounds are insoluble in water, they were dissolved previously in 1% DMSO and then added to the aqueous medium DMEM. The spectra were recorded for both complexes in the range of 260–460 nm, immediately and after 24 and 48h.

The comparison between the electronic spectra registered in DMSO (Fig. A and B) and in the DMEM medium (containing 1% DMSO) (Fig. C and D) indicates that there are changes in the spectral behavior suggesting interactions with the solvent and with the component of the biological medium, for both complexes. Complex **(3)** are stable on two solvents evaluated. In DMEM medium, significant changes in the spectrum were verified with time variation for complex **(4)**. The disappearance of the electronic transition after 24 h may have occurred as a result of the interaction of the compound with the components of the biological medium, as phenol red. No changes were observed in the electronic spectra of complex **(4)** in DMSO, which confirms its stability in this solvent.

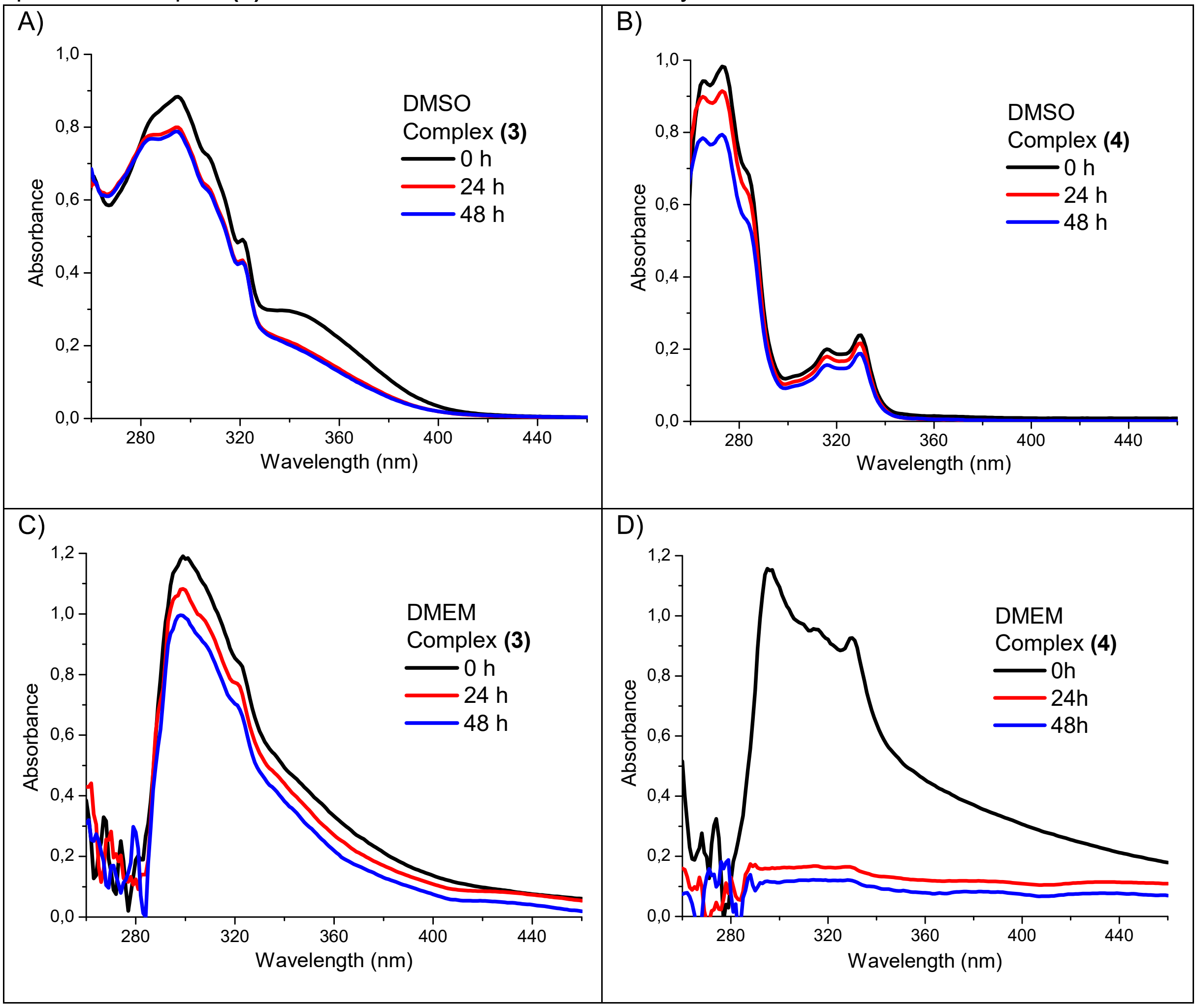

The conductivity measurements of solutions of complexes **(3)** and **(4)** in DMSO were obtained for fresh solutions (0 h), and after 24 and 48 h, in order to detect any changes in the structures of the complexes in solution, with time. The values obtained indicate that both complexes are neutral (Table 1). In DMSO, the range for 1:1 electrolyte is 0-46 μΩ/cm (reference W.J. Geary, The use of conductivity

measurements in organic solvents for the characterisation of coordination compounds, Coord. Chem. Rev. 7 (1971) 81, 122, doi:10.1016/S0010-8545(00)80009-0).
Thus, these complexes generate some neutral species in DMSO, suggesting that chlorine ligands aren't displaced from the coordination sphere of metallic centers, in both complexes, after 48h.

**Compound**	**Λ_m (μΩ/cm) 0 h**	**Λ_m (μΩ/cm) 24 h**	**Λ_m (μΩ/cm) 48 h**
Complex **(3)**	7.24	14.08	14.21
Complex **(4)**	2.08	3.75	3.96

Furthermore, ^{1}H NMR spectra were obtained for both complexes, in DMSO. Both complexes present identical spectrum since they are isomers. Here, we present the ^{1}H NMR spectrum just for complex **(4)**, which shows well defined signals typical of the ligand H2L2 (see Table and Fig. below). The signal at 2.50 ppm is attributed to solvent residual peak of DMSO, the signal at 2.50 ppm is attributed of H_2O and the signal at 5.80 is attributed to the solvent CH_2Cl_2.
In order to detect any changes in the structures of complex **(4)**, in DMSO, ^{1}H NMR spectra were obtained after 0, 24 and 48h. Thus, it can be concluded that the structure of compound **(4)** is maintained in solution, after 48h.

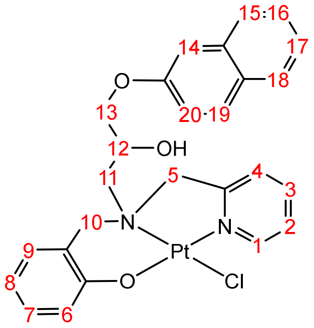

	**Multiplicity (J/Hz)**	**δH/ppm**
H1	*d* (*J* = 4)	8.53-8.50
H2, H3, H4	Multiplet	7.36-7.25
H5	Singlet	3.73
H6, H9	Multiplet	7.20-7.15
H7	Multiplet	7.14-7.09
H8, H14	Multiplet	6.79-6.72
H10, H13	Multiplet	4.02-3.87
H11	Multiplet	2.74-2.61
H12	Multiplet	3.82-3.77
H15, H19	Multiplet	7.78-7.72
H16, H17	Multiplet	7.47-7.41
H18	*d* (*J* = 1.8)	7.81-7.79
H20	*dd* (*J* = 8.8; 2.6)	7.05-7.02
-OH	Singlet	5.18

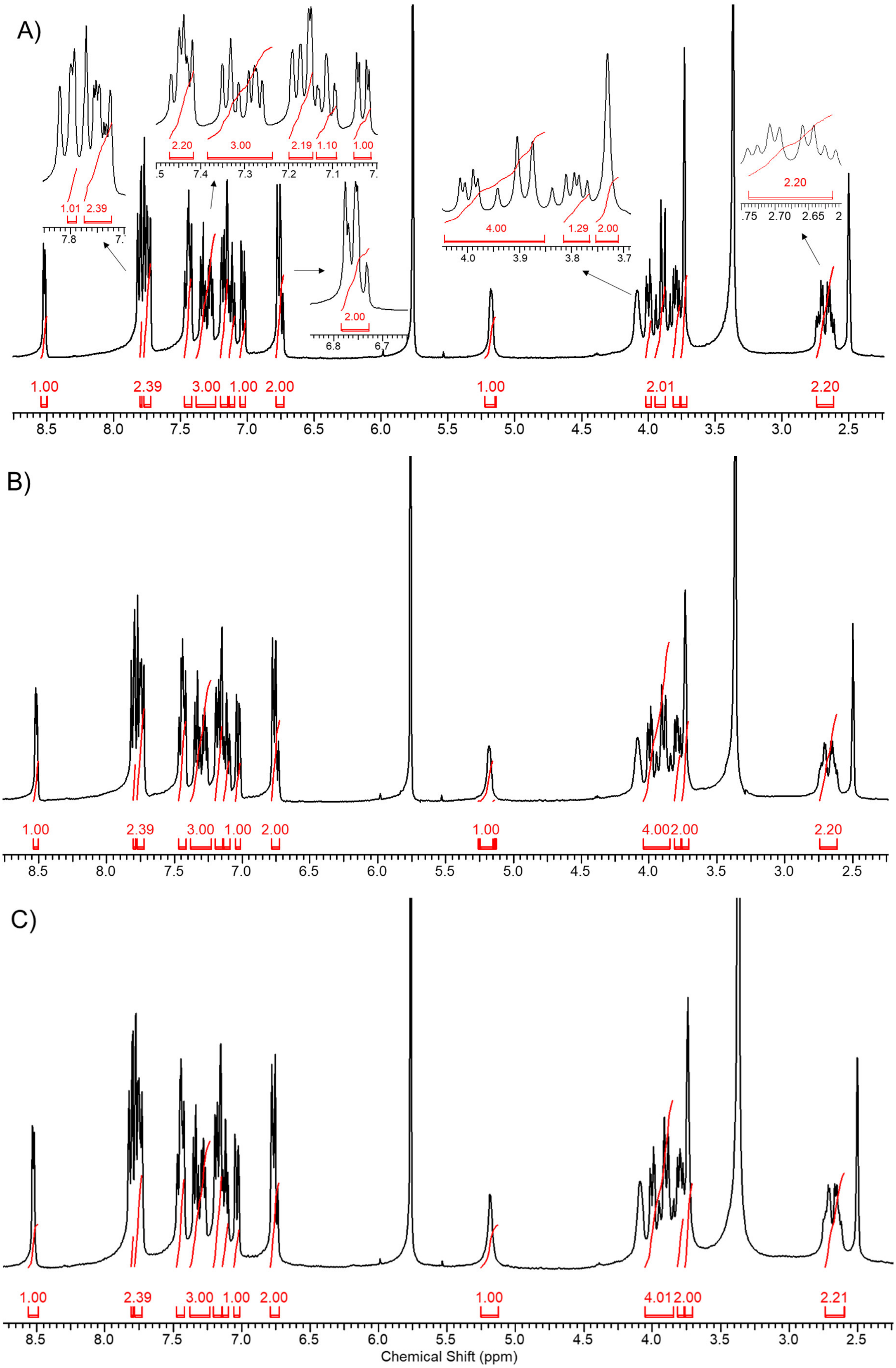

A)
B)
C)
1.00
2.39
3.00
1.00
2.00
1.00
2.01
2.20
1.01
2.20
2.19
1.10
4.00
1.29
4.002.00
4.012.00
2.21
Chemical Shift (ppm)
